# A machine learning approach to the development and prospective evaluation of a pediatric lung sound classification model

**DOI:** 10.1038/s41598-023-27399-5

**Published:** 2023-01-23

**Authors:** Ji Soo Park, Kyungdo Kim, Ji Hye Kim, Yun Jung Choi, Kwangsoo Kim, Dong In Suh

**Affiliations:** 1 Department of Pediatrics, Seoul National University College of Medicine, Seoul, South Korea; 2 Department of Biomedical Engineering, Duke University, Durham, NC USA; 3 Transdisciplinary Department of Medicine & Advanced Technology, Seoul National University Hospital, 101 Daehak-Ro, Jongno-Gu, Seoul, 03080 South Korea; 4 Department of Pediatrics, Seoul National University Hospital, Seoul National University College of Medicine, 101 Daehak-Ro, Jongno-Gu, Seoul, 03080 South Korea

**Keywords:** Respiratory signs and symptoms, Paediatric research

## Abstract

Auscultation, a cost-effective and non-invasive part of physical examination, is essential to diagnose pediatric respiratory disorders. Electronic stethoscopes allow transmission, storage, and analysis of lung sounds. We aimed to develop a machine learning model to classify pediatric respiratory sounds. Lung sounds were digitally recorded during routine physical examinations at a pediatric pulmonology outpatient clinic from July to November 2019 and labeled as normal, crackles, or wheezing. Ensemble support vector machine models were trained and evaluated for four classification tasks (normal vs. abnormal, crackles vs. wheezing, normal vs. crackles, and normal vs. wheezing) using K-fold cross-validation (*K* = 10). Model performance on a prospective validation set (June to July 2021) was compared with those of pediatricians and non-pediatricians. Total 680 clips were used for training and internal validation. The model accuracies during internal validation for normal vs. abnormal, crackles vs. wheezing, normal vs. crackles, and normal vs. wheezing were 83.68%, 83.67%, 80.94%, and 90.42%, respectively. The prospective validation (n = 90) accuracies were 82.22%, 67.74%, 67.80%, and 81.36%, respectively, which were comparable to pediatrician and non-pediatrician performance. An automated classification model of pediatric lung sounds is feasible and maybe utilized as a screening tool for respiratory disorders in this pandemic era.

## Introduction

Since the development of the first stethoscope by René Laennec in 1816, auscultation has been essential in the diagnosis of respiratory disorders^1^. Auscultation is quick, cost-effective, non-invasive, and radiation-free compared to other modes of diagnosis. Its role is especially important in children, who have more frequent respiratory infections and wheezing events than adults^2–5^. Auscultation with a stethoscope has certain limitations: it usually requires an in-person encounter, and lung sounds are prone to subjective interpretation and cannot be reviewed or shared between clinicians^6^. However, with the use of electronic stethoscopes, lung sounds can be stored, shared, and analyzed with various methods^7–9^.

The two most commonly noted abnormal lung sounds are wheezes and crackles. A wheeze is defined as a musical, high-pitched sound that can be heard upon inspiration and/or expiration, suggesting airway narrowing and airflow limitation. Wheezes typically appear as sinusoidal oscillations with sound energies in the range of 100 Hz to 1000 Hz, and lasting for longer than 80 ms. Crackles are short, explosive, nonmusical sounds, heard upon inspiration and sometimes during expiration, suggesting intermittent airway closure and opening of small airways. Crackles typically appear as rapidly dampened wave deflections, with typical frequencies and durations of 650 Hz and 5 ms, respectively, for fine crackles, and 350 Hz and 15 ms, respectively, for coarse crackles. Wheezing may be heard during bronchiolitis, asthma exacerbation, or other obstructive airway disorders, and crackles may suggest pulmonary edema, pneumonia, or interstitial lung diseases^10^.

Previous studies have been performed in the attempt to classify abnormal lung sounds by using machine learning techniques^11,12^. However, there have been few studies on pediatric lung sound classification; most studies involve detection of wheezing, which is the most distinctive adventitious lung sound^13,14^. Urban et al. have detected wheezing from overnight recordings of inpatients by sensitivity and specificity of 98%, and Gryzwalski et al. have detected abnormal lung sounds with mean F1 score of 0.625. Pediatric lung sounds differ from adult lung sounds in that the respiratory rates and heart rates vary widely according to age and are harder to obtain in children because of low cooperability. Although there are a few public lung sound databases, most datasets lack or include only a small number of pediatric lung sounds^15–17^.

In this study, we aimed to derive a machine learning model to classify pediatric electronic respiratory sounds from a database of real-world auscultation sounds collected from a pediatric respiratory clinic.

## Results

**Figure 1:**
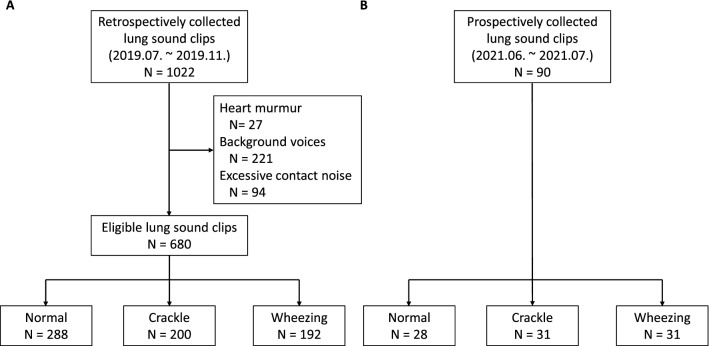
Study flowchart of the collection of lung sound data for (**A**) the training and internal validation set and (**B**) the prospective validation set.

In the training and internal validation set, a total of 1022 clips were collected; of these, 27 clips were excluded because of heart murmurs, 221 because of background conversation noise, and 94 because of excessive contact noise caused by movement of the stethoscope. A total of 680 lung sound clips were eligible for analysis, including 288 classified as normal, 200 as crackles, and 192 as wheezing sounds (Fig. 1). The prospective validation set included 90 consecutive clips collected during the prospective validation period (normal: 28, crackles: 31, wheezing: 31). The mean length of clips in the training and internal validation set was 4.1 ± 1.8 s and the mean length of clips in the prospective validation set was 4.0 ± 1.5 s. The mean respiratory rate was 30.2 and 27.2 breaths/min, respectively, in the two datasets (Table 1). The comparison between our datasets and the publicly available International Conference on Biomedical and Health Informatics (ICBHI) 2017 Challenge Respiratory Sound Database are show in Table 2 and in eTable 1.

**Table 1:** Data characteristics of lung sound clips in the training and internal validation set and the prospective validation set.

Training and internal validation set	Normal	Crackles	Wheezing	Total
	(n = 288)	(n = 200)	(n = 192)	(N = 680)
Length of clip (s)	4.7 ± 1.8	4.3 ± 1.7	3.1 ± 1.2	4.1 ± 1.8
**Number of breath cycles**				
1 cycle	3 (11.1%)	42 (21.0%)	64 (33.3%)	138 (20.3%)
2 cycles	256 (88.9%)	158 (79.0%)	128 (66.7%)	542 (79.7%)
Respiratory rate (/min)	27.3 ± 10.2	28.4 ± 12.3	36.4 ± 16.5	30.2 ± 13.4
**Heart sound**				
Inaudible	71 (24.7%)	49 (24.5%)	50 (26.0%)	170 (25.0%)
Normal	217 (75.3%)	151 (75.5%)	142 (74.0%)	510 (75.0%)

Prospective validation set	Normal	Crackle	Wheezing	Total
	(n = 28)	(n = 31)	(n = 31)	(N = 90)
Length of clip (s)	4.9 ± 1.6	4.0 ± 1.2	3.3 ± 1.2	4.0 ± 1.5
**Number of breath cycles**				
1 cycle	2 (7.1%)	13 (41.9%)	15 (48.4%)	30 (33.3%)
2 cycles	26 (92.9%)	18 (58.1%)	16 (51.6%)	60 (66.7%)
Respiratory rate (/min)	26.1 ± 8.5	24.6 ± 6.1	30.8 ± 13.0	27.2 ± 9.9
**Heart sound**				
Inaudible	3 (10.7%)	0 (0.0%)	2 (6.5%)	5 (5.6%)
Normal	25 (89.3%)	31 (100.0%)	29 (93.5%)	85 (94.4%)

**Table 2:** Composition of number of breath cycles in the current study data and the International Conference on Biomedical and Health Informatics (ICBHI) 2017 Challenge Respiratory Sound Database.

Dataset	Total	Normal	Wheeze	Crackles
**ICBHI 2017**				
Pediatric	782	642	75	65
**This study**	1312	599	367	407
Training set	1222	544	320	358
Test set	150	54	47	49

**Figure 2:**
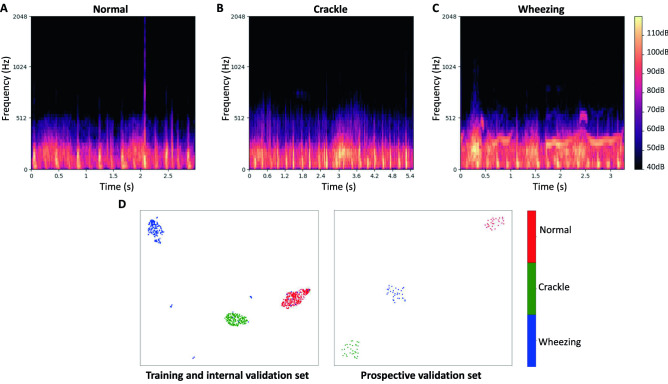
Typical mel-spectrograms labeled as normal (**A**), crackles (**B**), and wheezing (**C**), and UMAP visualization of lung sounds (**D**). (**A**) The typical normal lung sound mel-spectrogram exhibits homogeneous power intensity at ~ 200 Hz, mainly during inspiration and early expiration. (**B**) Crackles are characterized by multiple short, high-intensity bursts over a wide frequency, around 200–300 Hz, mainly during inspiration. (**C**) Wheezing is characterized by continuous musical sounds of narrow frequency bands, above 200 Hz. (**D**) The three classes of lung sounds are projected into three clusters by using UMAP for both the training and internal validation set and the prospective validation set. *UMAP* uniform manifold approximation and projection.

**Figure 3:**
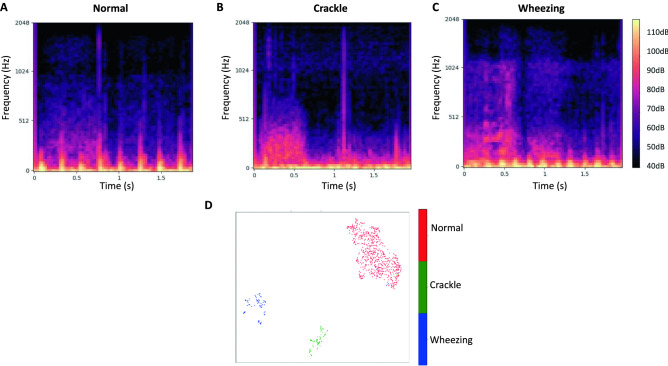
Mel-spectrogram and UMAP visualization of pediatric samples from publicly available lung sound dataset the International Conference on Biomedical and Health Informatics (ICBHI) 2017 Challenge Respiratory Sound Database. Mel-spectrograms labeled as normal (**A**), crackles (**B**), and wheezing (**C**), and UMAP visualization of lung sounds (**D**).

Typical mel-spectrograms of the three classes are presented in Fig. 2. The typical normal lung sound mel-spectrogram shows homogeneous power intensity in the ~ 200 Hz frequency mainly during inspiration and in early expiration. Crackle is characterized by multiple short high-intensity bursts over a wide frequency around 200–300 Hz, mainly during inspiration. Wheezing is characterized by continuous musical sounds of narrow frequency bands above 200 Hz. The 2D-plot of the training and internal validation data, generated by using UMAP, yielded clusters of each of the three classes. There was a degree of noise in the wheezing data, with wheezing subgroups overlapping with the crackle and normal groups. The 2D-plot of the prospective validation data yielded clear clustering of the three classes (Fig. 2). Mel-spectrograms and the UMAP presentation of the publicly available ICBHI 2017 pediatric data are presented in Fig. 3. Overall, the ICBHI pediatric data showed similar patterns as our data, but a few of the crackle and wheezing samples were mixed in the normal sample cluster.

Out of the machine learning models tested, the ensemble of SVMs produced best results (eTable 2). The machine learning model’s accuracy during K-fold cross-validation with the internal validation set for differentiation of normal vs. abnormal, crackles vs. wheezing, normal vs. crackles, and normal vs. wheezing were 83.68%, 83.67%, 80.94%, and 90.42%, respectively. The accuracy of the model during prospective validation with the set of 90 clips (28 normal, 31 crackles, and 31 wheezing) for those four tasks was 82.22%, 67.74%, 67.80%, and 81.36%, respectively. While the ICBHI pediatric data shows low recall due to severe imbalance between classes, other performance metrics were comparable to those of from our data (Table 3). Performance of other models tested on the ICBHI data is described in eTable 3.

**Table 3:** Model performance during internal validation, prospective validation, and external validation.

	Accuracy	Precision	Recall	F1-score
**Internal validation (N = 680)**				
Task 1: normal vs. abnormal	0.8368	0.8325	0.8340	0.8332
Task 2: crackles vs. wheezing	0.8367	0.8382	0.8360	0.8363
Task 3: normal vs. crackles	0.8094	0.8007	0.7938	0.7966
Task 4: normal vs. wheezing	0.9042	0.9053	0.8863	0.8936
**Prospective validation (N = 90)**				
Task 1: normal vs. abnormal	0.8222	0.7920	0.8122	0.8000
Task 2: crackles vs. wheezing	0.6774	0.6804	0.6774	0.6761
Task 3: normal vs. crackles	0.6780	0.6972	0.6849	0.6746
Task 4: normal vs. wheezing	0.8136	0.8298	0.8191	0.8127
**External validation (N = 782) (ICBHI 2017 pediatric data)**				
Task 1: normal vs. abnormal	0.835	0.649	0.171	0.793
Task 2: crackles vs. wheezing	0.764	0.828	0.707	0.764
Task 3: normal vs. crackles	0.911	1	0.031	0.871
Task 4: normal vs. wheezing	0.915	1	0.187	0.888

*ICBHI* International Conference on Biomedical and Health Informatics 2017 Challenge Respiratory Sound Database.

In the physician performance tests, the average accuracies of the pediatric infection and pulmonology specialists for the four tasks were 84.89%, 77.74%, 78.64%, and 84.41%, respectively. The average accuracies of physicians with other specialties were 80.67%, 66.13%, 73.90%, and 76.27%, respectively. The model performance was lower than that of pediatric specialists for all tasks, and higher than that of non-pediatric physicians in all tasks except for normal vs. crackles). None of the differences in performance between physicians and the model were statistically significant (Table 4). The precision, recall, and F1-scores of physicians for each task are described in the Supplementary Material eTable 4.

**Table 4:** Comparison of the accuracies of the model and 10 physicians for the prospective validation set.

	Model	Pediatric specialists	Other specialties	p-value^a^ (M vs P)	p-value^a^ (M vs O)
Task 1: normal vs. abnormal	0.8222	0.8489	0.8067	0.633	0.845
Task 2: crackle vs. wheezing	0.6774	0.7774	0.6613	0.128	0.922
Task 3: normal vs. crackle	0.6780	0.7864	0.7390	0.102	0.423
Task 4: normal vs. wheezing	0.8136	0.8441	0.7627	0.698	0.497

*M* model, *P* pediatric specialists, *O* physicians with other specialties.

^a^Chi-square test.

## Discussion

In this study, we developed and evaluated a machine learning model to classify pediatric electronic lung sounds by using real-world auscultation sounds collected from outpatient clinics. The model yielded a high accuracy of over 80% in all tasks during internal validation, with an accuracy of over 90% for normal vs. wheezing sounds. Upon prospective validation, the accuracy of the model for the four tasks decreased modestly, outperforming physicians other than pediatricians in three of the four tasks.

The classification performance of the model was highest for normal vs. wheezing sounds during internal validation, and for normal vs. abnormal sounds during prospective validation. Model performance was lowest for normal vs. crackle sounds during internal validation, and for crackle vs. wheezing sounds during prospective validation. Wheezing is a continuous musical sound with a distinct frequency band, produced by air flowing through a partially obstructed airway. Crackles are discontinuous noises with a wide range of frequencies, caused by intermittent airway closure and opening that is difficult to localize on a spectrogram. On the other hand, normal lung sounds, or vesicular sounds, are defined as non-musical, low-pass-filtered noises with a drop in energy at 200 Hz^10^. Therefore, it is easier to distinguish between wheezing and normal lung sounds than between wheezing or normal lung sounds and crackles, which is essentially a mixture of other lung sounds. This phenomenon was also demonstrated in the physicians’ performances, where crackles vs. wheezing and normal vs. crackle accuracies were lower than those of normal vs. abnormal and normal vs. wheezing for both pediatric and non-pediatric specialists. Past studies on machine learning-aided lung sound classification yielded high performance in in identifying wheezing in both adults and children^11–13^, but crackles and other adventitious sounds have rarely been successfully classified in children^14,18^.

The respiratory sounds of children are more challenging to collect and use for training, for various reasons. First, the respiratory rates of children vary widely according to age, compared to those in adults. In our training and internal validation dataset, the mean respiratory rate was 30.2 breaths/min, with a standard deviation of 13.4 breaths/min. This is a very wide range compared to the normal respiratory rate in adults (12 to 16 breaths/min). In addition, the smaller thoracic cage, relatively larger heart, and higher conductance of the chest wall in young children result in higher degrees of interference of heart sounds during chest auscultation than in adults^19,20^. Therefore, the majority of the recorded lung sounds will have heart sounds in the background. Finally, infants and young children are not cooperative during long sessions of auscultation and need constant soothing during physical examination, restricting the sufficient collection of clean respiratory sounds. In our study, we used a fair number of lung sounds obtained from children during routine physical examinations in the respiratory clinic, which allowed for the accurate classification of pediatric lung sounds despite these obstacles.

While it is harder to perform high-quality auscultation in children than in adults, the clinical significance of respiratory auscultation is more emphasized in children. Common respiratory disorders in children include respiratory infection, asthma, and foreign body aspiration^21,22^. Although radiologic diagnosis is generally easily accessible and highly accurate, they must be performed with discretion to minimize the radiation hazards, and there are still many parts of the world where radiologic tests are not readily available for children^23^. In addition, during viral pandemics, rapid, noninvasive screening and severity assessment of an individual’s respiratory state are essential^24^. In the Pneumonia Etiology Research for Child Health study, mortality from radiologic pneumonia was associated with different types of digitally recorded lung sounds^25^. Artificial intelligence (AI)-aided respiratory auscultation can help with diagnosis and prognostic prediction in children with respiratory disorders.

The ensemble model used in our study, based on SVMs, has the advantage of low computational costs while outperforming physicians other than pediatric pulmonology and infectious disease specialists. Recently, deep learning has yielded promising results in the medical domains where large volumes of data are generated, including lung sound classification tasks in adults. However, in children, where it is harder to obtain a large number of samples, there is a high possibility of overfitting, in addition to a high computational burden in the learning and inference process. By using SVM, which avoids problems with local minima during the learning process and avoids overfitting, our model can make efficient decisions with a modest amount of data^26^. In addition, the ensemble model provides the prediction probability of each SVM as output, which can be compared to aid physicians in the decision-making process. Finally, the model requires only features extracted from audio signals without any demographic or anthropometric information, which allows for easy applicability when loading the model on digital stethoscopes.

We have tried to overcome the ‘black-box’ phenomenon, common in machine learning, by applying explainable AI via UMAP. With UMAP, we were able to cluster the three classes of lung sounds—normal, crackles, and wheezing—on a 2D-plot. The clustering pattern was slightly different between the training and internal validation set and the prospective validation set, which were obtained two years apart. This is plausible as the patterns of practice of the recording physician constantly changes with time, and the physicians who edited and labeled the prospective set differed from those who labeled the training and internal validation set.

There are some limitations to our study. First, our datasets were limited in size and did not allow for deep learning inference. In addition, our study was based on a single-center cohort and recorded from a single recording device; therefore, the generalizability of our model for different devices and cohorts needs further validation. Third, we trained and tested our model for classification of three classes of lung sounds, when, in reality, there are many more types of adventitious sounds. Fourth, our SVM model uses a radial basis function kernel rather than a linear kernel to derive the best-performing model, making it difficult to retrospectively evaluate feature importance. Finally, our study showed feasibility to classify preprocessed breath cycles into different classes, but to apply this in practice, a preceding step to detect classifiable breath cycles from noise is essential. Nonetheless, we tested the generalizability of the model by performing a prospective validation. Further study with a larger sample would allow for more complex modeling and an improved performance.

In summary, we developed and prospectively evaluated a machine learning model for classification of electronically recorded pediatric lung sounds. The model yielded modest performance compared to pediatric pulmonology and infection specialists, and promising results compared to other specialists. In this pandemic era, AI-aided auscultation may improve the efficiency of clinical practice in pediatric patients.

## Methods

### Data source and labeling

**Figure 4:**
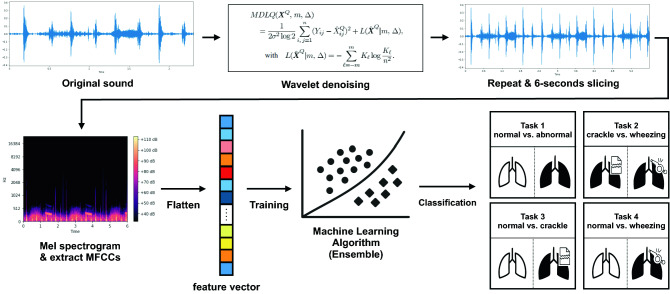
Data preprocessing and machine learning algorithm framework. The original sound clip underwent wavelet denoising, and was duplicated and cropped into 6-s windows. MFCCs extracted from the mel-spectrograms were used to train the ensemble models of the support vector machines. Four classification tasks were used for training: (1) normal vs. abnormal, (2) crackles vs. wheezing, (3) normal vs. crackles, and (4) normal vs. wheezing. MFCC, mel-frequency cepstral coefficient.

Lung sounds were recorded during routine physical examinations at the Pediatric Pulmonology outpatient clinic of Seoul National University Children’s Hospital by using a digital stethoscope (Thinklabs One Digital Stethoscope; Thinklabs Medical LLC, Centennial, CO, USA) connected to a wired audio recorder (PCM-A10, Sony, Tokyo, Japan), with a sampling rate of 44,100 Hz. Training and internal validation sets were recorded from July 2019 to November 2019, and prospective validation sets were recorded from June 2021 to July 2021. All lung sounds were recorded by a board-certified pediatric pulmonologist with 20 years of experience (D.I.S.). Audacity software (https://audacityteam.org/) was used to crop recorded lung sounds were into short clips containing one or two breath cycles. These sounds were classified as normal, crackles, and wheezes by pediatric pulmonologists (training and internal validation set: D.I.S., J.S.P.; prospective validation set: Y.J.C., J.H.K.). Wheezing was defined as inspiratory or expiratory musical sounds with frequencies of 100–1000 Hz that lasted longer than 80 ms. Crackles included both fine and coarse crackles, and were defined as explosive and repetitive short sounds (each 5–15 ms in length) of diffuse frequencies from 100 to 700 Hz that were heard during inspiration and/or expiration. Edited clips were included in the data sets if the two labeling physicians agreed on the label and excluded when there were heart murmurs louder than grade 2, background conversational noise, or contact noise that lasted for more than half of the breath cycle (Fig. 4).

The recording of electronic auscultation sounds and the machine learning analysis in this study were approved by the institutional review board of Seoul National University Hospital (No. H-1907-050-1047 and No. H-2201-076-1291). Informed consent was waived by the review board of Seoul National University Hospital as the recording of auscultation sounds was a routine part of the physical examinations, and no personal information other than lung sounds were collected. All research was performed in accordance with the Declaration of Helsinki.

### Data preprocessing and feature selection

Lung sound clips contain environmental sounds such as contact noise caused by friction between the stethoscope and skin or clothing, as well as ambient noise. We used the BayesShrink denoising method for the effective extraction of the desired lung sound^27^. The sound clips were cropped into 6-s windows: for clips longer than 6 s, only the first 6 s were used, and for clips shorter than 6 s, clips were duplicated until longer than 6 s, and cropped at 6 s. Our reasoning for the use of 6-s windows was as follows: the normal adult respiratory rate is around 12–16 breaths/min and the normal infant respiratory rate is 30–40 breaths/min; hence, 6 s would contain 1–4 breaths for all ages.

Mel-frequency cepstral coefficients (MFCCs) were used to extract acoustic features. MFCCs represent the power spectra of short sound frames according to the mel scale, a frequency scale that is familiar to the human auditory system. MFCCs are widely used for sound processing and analysis^28^. We extracted 40 MFCCs by using a fast-Fourier-transform window length of 660 samples, hop length of 512 samples, and Hann windowing.

There are several methods in digital sound processing that are applied in using sound files in machine learning; some studies use the raw sound, others have extracted frequency domain information through Fourier Transform (FT), and some previous research uses the log-mel features^9,29,30^. Among these, we chose MFCC features as an input to the model because our raw sound was collected in a real environment, containing numerous unnecessary noises. In the case of FT, temporal information is not adequately reflected in the features. Log-Mel spectrogram was not used in this case because the dimension would be too high compared to the number of samples.

### Mel-spectrogram visualization and UMAP embedding

For pre-modeling explainability and exploratory data analysis, we visualized individual sound clips into mel-spectrograms. A mel-spectrogram is a visualization of the frequency spectrum of an audio signal over time, where the frequency axis is filtered into the mel scale. We used the same hyperparameters as those for MFCC extraction.

Uniform manifold approximation and projection (UMAP) is a dimension-reduction technique that allows the two-dimensional (2D) visualization of data while preserving the global structure and local relationships within the data^31^. We applied UMAP to the training and internal validation set as well as the prospective validation set, with the following parameters: number of neighbors = 20, minimum distance = 0.3, distance metric = ‘cosine’.

### Comparison of data with existing pediatric lung sound database

To compare the current study data with the available lung sound database, we used the International Conference on Biomedical and Health Informatics (ICBHI) 2017 Challenge Respiratory Sound Database^32^. This public database contains lung sounds from all ages including some pediatric samples. We examined the distribution of normal, wheeze, and crackle in the pediatric samples from the ICHBI database. Also, we visualized sound clips from the ICBHI database as mel-spectrograms. Finally, we applied UMAP to the pediatric samples in ICBHI.

### Machine learning modeling

With MFCCs as inputs, we created an ensemble model based on a support vector machine (SVM). The ensemble model based on SVM was chosen after comparison of simple SVM, random forest, Gaussian process, and ensemble of SVM models (Detailed method in Supplementary Material). A SVM is a lighter model compared to deep learning models that use neural networks and has the advantage of determining a robust decision boundary without overfitting when the sample size is small. The ensemble method is a machine learning methodology that combines predictions from multiple models to overcome overfitting and increase robustness. In this study, we designed an ensemble model by using a majority voting algorithm from 1 to 10 SVM models, in which the optimal number of SVM models and the type of kernel function were decided empirically.

Four classification tasks were carried out: (1) normal vs. abnormal, (2) crackle vs. wheezing, (3) normal vs. crackle, and (4) normal vs. wheezing. We used a single SVM model for classification of normal vs. abnormal, 4 models each for crackle vs. wheezing and normal vs. wheezing, and 10 models for normal vs. crackle. A radial basis function kernel was used. The overall data processing and model pipeline is illustrated in Fig. 1.

### Training and internal validation

K-fold cross-validation (*K* = 10) was used for training and internal validation. Cross-validation is widely used in machine learning to prevent overfitting while using all available data as training and validation sets. The training dataset is split into *K* smaller sets, or ‘folds’, a model is trained by using *K* − 1 of the folds as training data, and the accuracy of the resulting model is validated on the remaining part of the data. The procedure is repeated *K* times, with each *K* fold being used once for validation. In our study, we used a stratified K-fold approach, where data is split into *K* folds that all contain the same proportion of labeled classes.

The performance of the model was evaluated by using accuracy, precision, recall, and F1-score, defined as follows: accuracy = (true positives [TP] + true negatives [TN])/(TP + false positives [FP] + false negatives [FN] + TN), precision = TP/(TP + FP), recall = TP/(TP + FN), and F1-score = harmonic mean of precision and recall.

### Prospective validation, external validation and physician performance

The model was evaluated by using a prospectively collected validation dataset (June 2021 to July 2021). The prospective validation set was obtained in a consecutive manner with a target number of 29–31 samples for each class and 90 samples in total. Two independent researchers edited and labeled the prospectively collected auscultation sounds, and the first 29–31 samples for each class were included without selection. After performing the same data preprocessing, the same K-fold cross-validation method was applied, which provided an ensemble model for each fold. For prospective validation, a nested cross-validation model was applied, where an overall ensemble model was created based on majority voting including all SVM models in ensembles for each of the *K* folds^33^. Model performance was evaluated by using accuracy, precision, recall, and F1-score, as in the internal validation. External validation on the ICBHI pediatric data was also done.

Physician performance tests were conducted by using the prospective validation dataset to compare the lung sound classification performance of the machine learning model with that of physicians. A total of 10 physicians participated: 5 pediatric specialists (3 pediatric pulmonologists and 2 pediatric infectious disease specialists) with 6 to 8 years of clinical experience (performing auscultation daily), and 5 non-pediatric specialists who did not routinely perform auscultation. The average accuracies of the pediatricians and non-pediatricians in terms of the four classification tasks were calculated and compared to the model’s accuracy by using the chi-square test.

### Software

Continuous data are presented as the mean ± standard deviation, and categorical data are presented as frequencies (percentages). Python (ver. 3.8; www.python.org) was used for data preprocessing and machine learning. The Librosa package (ver. 0.8.1) was used, as well as the BayesShrink method with soft thresholding in scikit-image (ver. 0.19.0), for data preprocessing, including denoising. The SVM classifier and StratifiedKfold modules in the scikit-learn package (ver. 1.0.2) were used. For comparison of model and physician performance in classifying the prospective validation set, chi-square tests were performed with R statistical software (ver. 4.0.3; R Foundation for Statistical Computing, Vienna, Austria).

### Ethics approval and consent to participate

The recording of electronic auscultation sounds and the machine learning analysis in this study were approved by the institutional review board of Seoul National University Hospital (No. H-1907-050-1047 and No. H-2201-076-1291). Informed consent was waived by the review board as the recording of auscultation sounds was a routine part of the physical examinations, and no personal information other than lung sounds were collected.

## Supplementary Information

Supplementary Information.

## Data Availability

The datasets used and/or analyzed during the current study are available from the corresponding author on reasonable request.

## Abbreviations

AI: Artificial intelligence
FN: False negatives
FP: False positives
MFCC: Mel-frequency cepstral coefficient
SVM: Support vector machine
TN: True negatives
TP: True positives
UMAP: Uniform manifold approximation and projection

## Code availability

Codes used for the current study are available at https://github.com/Medical-K/pediatric_lungsound.
