# Supplementary material for: A machine learning approach to the development and prospective evaluation of a pediatric lung sound classification model
Source: Sci Rep. 2023 Jan 23;13:1289. doi: 10.1038/s41598-023-27399-5 (PMC9871007; doi:10.1038/s41598-023-27399-5)
Supplement: Supplementary file 1 — Supplementary Information. [file 41598_2023_27399_MOESM1_ESM.docx]

**- Supplementary Material -**

**A machine learning approach to the development and prospective evaluation of a pediatric lung sound classification model**

**CONTENTS**

| **eTable 1.** Respiratory rates of the current study data and the International Conference on Biomedical and Health Informatics 2017 Challenge Respiratory Sound Database. | **… Page 2** |
| --- | --- |
| **eTable 2.** Performance of different machine learning models on the internal validation set. | **… Page 3** |
| **eTable 3.** Performance of different machine learning models on an external dataset. | **… Page 4** |
| **eTable 4.** Performance metrics of pediatric specialists and other specialists. | **… Page 5** |
| **Supplementary Text.** Methods: Machine learning modeling – Use of the support vector machine ensemble | **… Page 6** |
| **References for Supplementary Material** | **… Page 8** |

**eTable 1.** Respiratory rates of the current study data and the International Conference on Biomedical and Health Informatics (ICBHI) 2017 Challenge Respiratory Sound Database.

| **Dataset** | **Normal** | **Wheeze** | **Crackles** | **Total** |
| --- | --- | --- | --- | --- |
| **ICBHI 2017** | 30.3 ± 21.7 | 27.5 ± 18.0 | 24.7 ± 11.6 | 27.8 ± 18.5 |
| **- Adult** | 27.6 ± 18.3 | 26.3 ± 15.6 | 24.4 ± 11.1 | 26.1 ± 15.6 |
| **- Pediatric** | 42.9 ± 30.3 | 41.5 ± 32.0 | 34.7 ±19.3 | 42.0 ± 29.7 |
| **This study** |  |  |  |  |
| **- Training set** | 27.3 ± 10.2 | 36.4 ± 16.5 | 28.4 ± 12.3 | 30.2 ± 13.4 |
| **- Test set** | 26.1 ± 8.5 | 30.8 ± 13.0 | 24.6 ± 6.1 | 27.2 ± 9.9 |

**eTable 2.** Performance of different machine learning models on the internal validation set.

|  | **Accuracy** | **Precision** | **Recall** | **F1 score** |
| --- | --- | --- | --- | --- |
| **Task 1: Normal vs. Abnormal** | | | | |
| **SVM** | **0.8368** | **0.8325** | **0.8340** | **0.8332** |
| **Random Forest** | 0.7632 | 0.7505 | 0.8827 | 0.7567 |
| **Gaussian Process** | 0.7984 | 0.8400 | 0.8035 | 0.7992 |
| **Ensemble** | 0.8294 | 0.8575 | 0.8444 | 0.8296 |
| **Task 2: Crackles vs. Wheezing** | | | | |
| **SVM** | **0.8368** | **0.8325** | **0.8340** | **0.8332** |
| **Random Forest** | 0.7632 | 0.7505 | 0.8827 | 0.7567 |
| **Gaussian Process** | 0.7984 | 0.8400 | 0.8035 | 0.7992 |
| **Ensemble** | 0.8294 | 0.8575 | 0.8444 | 0.8296 |
| **Task 3: Normal vs. Crackles** | | | | |
| **SVM** | 0.7951 | 0.7778 | 0.7000 | 0.7931 |
| **Random Forest** | 0.7643 | 0.7607 | 0.6200 | 0.7594 |
| **Gaussian Process** | 0.7828 | 0.7423 | 0.7200 | 0.7822 |
| **Ensemble** | **0.8094** | **0.8007** | **0.7938** | **0.7966** |
| **Task 4: Normal vs. Wheezing** | | | | |
| **SVM** | 0.8979 | 0.9281 | 0.8073 | 0.8965 |
| **Random Forest** | 0.8333 | 0.8889 | 0.6667 | 0.8278 |
| **Gaussian Process** | 0.8563 | 0.8868 | 0.7343 | 0.8534 |
| **Ensemble** | **0.9042** | **0.9053** | **0.8863** | **0.8936** |

**eTable 3.** Performance of different machine learning models on an external dataset.

|  | **Accuracy** | **Precision** | **Recall** | **F1 score** |
| --- | --- | --- | --- | --- |
| **Task 1: Normal vs. Abnormal** | | | | |
| **SVM** | **0.84** | **0.727** | **0.171** | **0.797** |
| **Random Forest** | 0.835 | 1 | 0.079 | 0.772 |
| **Gaussian Process** | 0.834 | 0.562 | 0.321 | 0.815 |
| **Ensemble** | 0.835 | 0.649 | 0.171 | 0.793 |
| **Task 2: Crackles vs. Wheezing** | | | | |
| **SVM** | 0.757 | 0.781 | 0.76 | 0.757 |
| **Random Forest** | 0.743 | 0.783 | 0.72 | 0.743 |
| **Gaussian Process** | 0.464 | 0 | 0 | 0.294 |
| **Ensemble** | **0.764** | **0.828** | **0.707** | **0.764** |
| **Task 3: Normal vs. Crackles** | | | | |
| **SVM** | 0.908 | 0 | 0 | 0.864 |
| **Random Forest** | 0.907 | 0 | 0 | 0.864 |
| **Gaussian Process** | 0.896 | 0.25 | 0.062 | 0.867 |
| **Ensemble** | **0.911** | **1** | **0.031** | **0.871** |
| **Task 4: Normal vs. Wheezing** | | | | |
| **SVM** | 0.914 | 0.882 | 0.2 | 0.888 |
| **Random Forest** | 0.911 | 0.923 | 0.16 | 0.881 |
| **Gaussian Process** | 0.915 | 0.733 | 0.293 | 0.898 |
| **Ensemble** | **0.915** | **1** | **0.187** | **0.888** |

**eTable4.** Performance metrics of pediatric specialists and other specialists

| **Pediatric Specialists (N=5)** | **Accuracy** | **Precision** | **Recall** | **F1-score** |
| --- | --- | --- | --- | --- |
| **Task 1: Normal vs. Abnormal** | 0.8489 | 0.9291 | 0.8452 | 0.8851 |
| **Task 2: Crackles vs. Wheezing** | 0.7774 | 0.7584 | 0.8710 | 0.8108 |
| **Task 3: Normal vs. Crackles** | 0.7864 | 0.8750 | 0.7226 | 0.7915 |
| **Task 4: Normal vs. Wheezing** | 0.8441 | 0.9699 | 0.8323 | 0.8958 |
| **Other Specialists (N=5)** | **Accuracy** | **Precision** | **Recall** | **F1-score** |
| **Task 1: Normal vs. Abnormal** | 0.8067 | 0.9208 | 0.8133 | 0.8637 |
| **Task 2: Crackles vs. Wheezing** | 0.6613 | 0.6543 | 0.6839 | 0.6688 |
| **Task 3: Normal vs. Crackles** | 0.7390 | 0.8250 | 0.6387 | 0.7200 |
| **Task 4: Normal vs. Wheezing** | 0.7627 | 0.9381 | 0.6839 | 0.7910 |

**SUPPLEMENTARY TEXT**

**METHODS**

**Machine learning modeling – Use of the support vector machine**

SVM is based on the theory of convex programming, a simple and powerful idea to solve a high-dimensional convergence problem.^1^ SVM is mainly used in clinical research because of its low computational cost, global optimization, strong adaptability, and good generalization ability.^2,3^ Especially in ensemble learning, based on the theory that multiple weaker models become stronger when combined, the light and simple model like SVM can be more powerful. Many previous studies used SVM as a basis model for ensemble learning.^4,5^ In this research, we first trained each SVM model differently and predicted each case. After that, we combined the prediction and made a majority voting for the final prediction. We followed this process in both internal validation and external validation.

In this research, we have tried numerous machine learning models for the dataset. There were random forest, Gaussian process,^6,7^ XGBoost, and deep neural networks. However, these methods were not successful or showed inferior performance to SVM. We have found some plausible reasoning in literature. First, the random forest model can be biased in some datasets.^8,9^ This is because even the random forest randomly selected the tree made by different features; if the overall data is not well distributed in the center, the result of the tree can be biased. XGboost is a more advanced model to avoid overfitting using a boosting algorithm. However, XGboost is still unsuitable for categorical values and needs a long time for training and prediction.^10^ Because we have extracted 40 values from the continuous raw sound, we need a more compact model that can fit well into our data. A deep neural network is a deep learning model that connects multiple perceptron networks. It is a simple but powerful model that links many layers in one model. However, if the feature is sparse and the number of data is small, the performance drops significantly.^11^ More specifically, when it comes to clinical data like our data, sample number is relatively small and feature is hard to extract, whereas the data is very high-dimension.^12,13^ This is because a number of clinical data is from the real world, which has numerous noise and measurement errors. Therefore, much clinical research still does not use a deep neural network but uses a classical machine learning model like this research.

**Details of the SVM model**

Normal vs Abnormal

model_1 = SVC(gamma= 1/n_features, kernel='rbf', C=1)

Crackle vs Wheezing & Normal vs Wheezing

model_1 = SVC(gamma= 1/n_features, kernel='rbf', C=1)

model_2 = SVC(gamma= 1/n_features, kernel='rbf', C=1.5)

model_3 = SVC(gamma= 1/n_features, kernel='rbf', C=0.5)

model_4 = SVC(gamma= 1/n_features, kernel='rbf', C=0.8)

Normal vs Crackle

model_1~10 = SVC(gamma= 1/n_features, kernel='rbf', C=0.2~2, increase by 0.2)

**References for Supplementary Material**

1 Anguita, D., Ghio, A., Greco, N., Oneto, L. & Ridella, S. Model selection for support vector machines: Advantages and disadvantages of the Machine Learning Theory. *he 2010 International Joint Conference on Neural Networks (IJCNN)*, 1-8, doi:10.1109/IJCNN.2010.5596450 (2010 ).

2 Fraiwan, L. *et al.* Automatic identification of respiratory diseases from stethoscopic lung sound signals using ensemble classifiers. *Biocybernetics and Biomedical Engineering* **41**, 1-14, doi:10.1016/j.bbe.2020.11.003 (2021).

3 Sen, I., Saraclar, M. & Kahya, Y. P. Differential Diagnosis of Asthma and COPD Based on Multivariate Pulmonary Sounds Analysis. *IEEE Trans Biomed Eng* **68**, 1601-1610, doi:10.1109/TBME.2021.3049288 (2021).

4 Mahesh, T. R., Vinoth Kumar, V., Vivek, V., Karthick Raghunath, K. M. & Sindhu Madhuri, G. Early predictive model for breast cancer classification using blended ensemble learning. *International Journal of System Assurance Engineering and Management*, doi:10.1007/s13198-022-01696-0 (2022).

5 Yang, Y. *et al.* Classification of Parkinson's disease based on multi-modal features and stacking ensemble learning. *J Neurosci Methods* **350**, 109019, doi:10.1016/j.jneumeth.2020.109019 (2021).

6 Liu, Zitao, Lei Wu, and Milos Hauskrecht. Modeling clinical time series using gaussian process sequences. *Proc 2013 SIAM International Conference on Data Minin*. Society for Industrial and Applied Mathematics. (2013).

7 Futoma J, Hariharan S, Heller K, Sendak M, Brajer N, Clement M, Bedoya A, O’brien C. An improved multi-output gaussian process rnn with real-time validation for early sepsis detection. *InMachine Learning for Healthcare Conference* 143-254, (2017)

8 Mendez, G. & Lohr, S. Estimating residual variance in random forest regression. *Computational Statistics & Data Analysis* **55**, 2937-2950, doi:10.1016/j.csda.2011.04.022 (2011).

9 Shah, A. D., Bartlett, J. W., Carpenter, J., Nicholas, O. & Hemingway, H. Comparison of random forest and parametric imputation models for imputing missing data using MICE: a CALIBER study. *Am J Epidemiol* **179**, 764-774, doi:10.1093/aje/kwt312 (2014).

10 Cao, D., Ma, Y., Sun, L. & Gao, L. Fast observation simulation method based on XGBoost for visible bands over the ocean surface under clear-sky conditions. *Remote Sensing Letters* **12**, 674-683, doi:10.1080/2150704x.2021.1925371 (2021).

11 Zohuri, B. Deep Learning Limitations and Flaws. *Modern Approaches on Material Science* **2**, doi:10.32474/mams.2020.02.000138 (2020).

12 Bandyk, M. G., Gopireddy, D. R., Lall, C., Balaji, K. C. & Dolz, J. MRI and CT bladder segmentation from classical to deep learning based approaches: Current limitations and lessons. *Comput Biol Med* **134**, 104472, doi:10.1016/j.compbiomed.2021.104472 (2021).

13 Shickel, B., Tighe, P. J., Bihorac, A. & Rashidi, P. Deep EHR: A Survey of Recent Advances in Deep Learning Techniques for Electronic Health Record (EHR) Analysis. *IEEE J Biomed Health Inform* **22**, 1589-1604, doi:10.1109/JBHI.2017.2767063 (2018).
